# An exception that proves the rule: recurrence free survival five years after extrapleural pneumonectomy for malignant pleural mesothelioma

**DOI:** 10.1186/s13019-014-0181-x

**Published:** 2014-11-18

**Authors:** Tom Treasure, Fergus Macbeth

**Affiliations:** Clinical Operational Research Unit, University College London, London, WC1H 0BT UK; Wales Cancer Trials Unit, Cardiff University, Cardiff, CF14 7XL UK

**Keywords:** Mesothelioma, Surgery, Extrapleural pneumonectomy

## Abstract

Are case reports at all relevant and useful? A case report of an unusual case of mesothelioma prompts a discussion and concludes that they do have a role but that their observations and conclusions need to be treated with care.

What can we learn from a clinical anecdote [1]? In the personal practice of individual doctors the rare unusual experiences often loom larger and influence our behaviour more perhaps than they ought. The bleed in an anti-coagulated patient is more vividly remembered than the unseen patients whose thromboembolic stroke has been prevented [2]. Nevertheless case reports of striking and unusual clinical cases, such as this one, are the bread and butter of many medical journals and it is perhaps worth considering the purposes they serve. In the era of evidence based healthcare when the randomised trial appears to be the ultimate arbiter of good practice, do they still earn their page space and PubMed citations?

Clearly for very rare conditions they do, as they may be the only source of evidence to guide patient management. For instance adult pulmonary blastoma has generated 20 or so case report PubMed citations in the past 10 years and the cumulated experience reported in them, and summarised in a handful of reviews, may help guide an uncertain clinical team - though even that function may now be superseded by online discussion forums and their more immediate feedback. But case reports of rare presentations or very unusual clinical manifestations of more common conditions are perhaps less helpful.

The report published in this journal [1] of an unusual patient with malignant pleural mesothelioma (MPM) with long term disease control following surgery alone is a case in point. Is it of mere passing interest or does it help us understand the condition and its management? The authors describe a single case with an uncharacteristic pattern of disease and their two conclusions that the long survival observed could be either attributed to 'margin-free tumor resection' or to the fact that some subgroups have `lower malignant potential, leading to improved survival' are entirely reasonable.

In patients with a typical MPM having radical surgery, the tumour always crosses the resection margin [3]. This presentation as a localised (albeit large) chest wall tumour is the exception; the characteristic disease along the visceral pleura was not present in this patient. For virtually all patients with mesothelioma the term 'macroscopic complete resection' is misleading. The definition of complete resection is histological. Look under the microscope and there is always disease up to the resection margin - but not in this case. It is the quite exceptional behaviour of the cancer in this instance which makes it worth reporting. It is a nice example of 'the exception proves the rule'. That there is an exception does not undermine the generality of the rule.^1^

We know only too well that all cancers, including mesothelioma are variable in their clinical behaviour and although histopathological examination (and now genomic testing) can provide clinically useful sub-classification, it is never a reliable predictor of outcome. Often regarded as the 'gold standard', histopathological diagnosis has nevertheless plenty of instances of exception and continues to be a negotiated 'framing' of the disease [4]. The 'frame' is shifted to accommodate new groupings and subdivisions. Histopathological diagnoses are not God-given and immutable but resolved by human committees.

So this case report does not really help us manage better the great majority of patients with mesothelioma. But it does remind us that each patient is unique and sometimes we need to consider a unique approach. In clinical surgery there are plenty of circumstances where the surgeon and patient agree a 'one-off' surgical plan and so they should. General rules are applied with judgement to reach an individualised decision. Good quality observational studies and data analyses based on them are often sufficient to provide the rule. That applies when there is a clear mechanistic and temporal relationship between the intervention and benefit. Common examples in thoracic surgery are the relief of tension pneumothorax, draining pus from an empyema, or retrieving an inhaled object from the airway. When there is long time course, variability in the cancer biology, and multiple treatments are used in sequence or in parallel, as in mesothelioma, it may require a randomised trial, so difficult to do in surgery [5], to clinch the argument [6]. MARS is widely accepted as putting EPP beyond reasonable practice in all but an exceptional case such as this. Even then the principle reason for survival may be that there was a 'lower malignant potential' as the authors suggest, rather than the surgery itself.

In summary, case reports are written and published when there is something exceptional to write up. In this instance it is a rare exception that merits publication and it indicates the existence of a general rule. Read with interest, treat with care!

## Endnotes

^1^http://en.wikipedia.org/wiki/Exception_that_proves_the_rule.
